# Diagnostic and treatment delay, quality of life and satisfaction with care in colorectal cancer patients: a study protocol

**DOI:** 10.1186/1477-7525-11-117

**Published:** 2013-07-11

**Authors:** Salvador Pita-Fernández, Sonia Pértega-Díaz, Beatriz López-Calviño, Teresa Seoane-Pillado, Esther Gago-García, Rocío Seijo-Bestilleiro, Paloma González-Santamaría, Alejandro Pazos-Sierra

**Affiliations:** 1 Clinical Epidemiology and Biostatistics Unit, Complexo Hospitalario Universitario A Coruña (CHUAC). SERGAS. Universidade de A Coruña, Hotel de Pacientes 7a Planta, As Xubias 84, A Coruña 15006, Spain; 2 Intensive Care Unit, Complexo Hospitalario Universitario A Coruña (CHUAC). SERGAS, As Xubias 84, A Coruña 15006, Spain; 3 Department of Information and Communication Technologies, Computer Science Faculty, University of A Coruña, Campus de Elviña s/n, A Coruña 15071, Spain

**Keywords:** Colorectal neoplasms, Quality of life, Patient satisfaction

## Abstract

**Background:**

Due to recent improvements in colorectal cancer survival, patient-reported outcomes, including health-related quality of life and satisfaction with care, have become well-established endpoints to determine the impact of the disease on the lives of patients.

The aim of this study is to determine prospectively, in a cohort of colorectal cancer incident cases: a) health-related quality of life, b) satisfaction with hospital-based care, and c) functional status. A secondary objective is to determine whether diagnostic/therapeutic delay influence quality of life or patients’ satisfaction levels.

**Methods/design:**

Single-centre prospective follow-up study of colorectal cancer patients diagnosed during the period 2011–2012 (n = 375).

This project was approved by the corresponding ethics review board, and informed consent is obtained from each patient. After diagnosis, patients are interviewed by a trained nurse, obtaining information on sociodemographic characteristics, family history of cancer, first symptoms, symptom perception and reaction to early symptoms. Quality of life is assessed with the EORTC QLQ-C30 and QLQ*-*CR29 questionnaires, and patients’ satisfaction with care is determined using the EORTC IN-PATSAT32. Functional status is measured with the Karnofsky Performance Status Scale.

Clinical records are also reviewed to collect information on comorbidity, tumour characteristics, treatment, hospital consultations and exploratory procedures.

Symptoms-to-diagnosis interval is defined as the time from the date of first symptoms until the cytohistological confirmation of cancer. Treatment delay is defined as the time between diagnosis and surgical treatment.

All the patients will be followed-up for a maximum of 2 years. For survivors, assessments will be re-evaluated at one and two years after the diagnosis.

Multiple linear/logistic regression models will be used to identify variables associated with the patients’ functional status, quality of life and satisfaction with care score. Changes in quality of life over time will be analysed with linear mixed-effects regression models.

**Discussion:**

The results will provide a deeper understanding of the impact of colorectal cancer from a more patient-centred approach, allowing us to identify groups of patients in need of additional attention, as well as areas for improvement. Special attention will be given to the relationship between diagnostic/therapeutic delay and patients’ quality of life and satisfaction with the care received.

## Background

Colorectal cancer remains a significant cause of morbidity and mortality throughout the world. It is the third most commonly diagnosed cancer in males and the second in females, with over 1.2 million incident cases and 608,700 deaths estimated to have occurred in 2008 [1]. In recent years, colorectal cancer death rates have decreased in several Western countries [2], a result of screening policies, the reduction of risk factors and improved treatment [3].

Estimates of long-term cancer survival rates in Europe, published by the EUROCARE-4 project, show a five-year relative survival of colorectal cancer patients ranging from 64% in Switzerland to 44% in Poland [4]. In Spain, this figure stands at 61.5%. Ten-year relative survival is only about 5 percentage points lower, which indicates a very good prognosis for those patients who are still alive after five years in all countries.

Because of these recent improvements in survival, patient-reported outcomes, including health-related quality of life (QOL) and satisfaction with care, have become well-established outcomes in cancer patients, along with traditional endpoints of tumour response and survival [5]. Patients with cancer may also exhibit functional impairment as a direct result of the disease or treatment-related sequelae [6]. Moreover, a positive relationship between quality of life data and the survival of cancer patients was established [7].

However, despite its importance, quality of life is not routinely measured in the care of patients with cancer. One of the difficulties lies in choosing the appropriate instrument to measure patients’ quality of life. Questionnaires developed by the European Organisation for Research and Treatment of Cancer (EORTC) [8,9] and the FACT-C (Functional Assessment of Cancer Therapy-Colorectal) [10] have become the most used in research studies.

Several studies have been published in the last few years analysing early [11-18] and long-term [19-28] quality of life in colorectal cancer patients. In general, these studies show that QOL deteriorates early after treatment but gradually improves if there is no recurrence or progression of the disease. In the long-term, the QOL of colorectal cancer survivors appears to be comparable to the general population. However, survivors have a slightly worse physical QOL, suffer from bowel symptoms and cancer-related distress, and have worse depression scores [29]. Some factors, such as higher age, a lower income or a higher number of comorbidities are clearly associated with lower levels of QOL scores. However, results regarding the effects of other cancer-related factors, such as the presence of a stoma, stage or treatment regimens seem to be inconclusive [29].

On the other hand, the assessment of patient satisfaction has also been recognised as a key indicator of health care quality, especially in oncology, where patients are subjected to increasingly complex treatments, exhaustive follow-ups, and numerous visits to hospital. It has been demonstrated that measurement of patient satisfaction requires the use of multi-item scales to address the different dimensions of satisfaction. The European Organisation for Research and Treatment of Cancer (EORTC) Quality of Life Group recently developed the EORTC INPATSAT32, a questionnaire to assess the perception cancer patients have of the quality of their hospital-based care [30]. The INPATSAT32 is an instrument that makes it possible to measure patients’ perception of the quality of medical care, nursing care and care organisation and services received in the hospital. It has been developed and tested in a cross-cultural context, therefore allowing for comparisons among several hospitals or health systems in different countries. The psychometric properties of the Spanish version of the INPATSAT32 have been investigated in 80 cancer patients with different tumour sites [31], but to the best of our knowledge there are no papers determining satisfaction with care exclusively in colorectal cancer patients.

Over the last few years, our group has participated in two multicentre projects in colorectal cancer patients. We have been investigating whether diagnosis and therapeutic delay, as well as different follow-up strategies, are associated with a higher survival rate in colorectal cancer patients [32,33]. Since studies in other cancer locations suggest that a long total delay may influence QOL and survival [34], in this project we aim to investigate in greater detail the association between diagnostic delay, treatment delay and follow-up strategies with subsequent quality of life and colorectal cancer patients’ satisfaction scores.

### Objectives

#### Main objectives

To determine the following in colorectal cancer incident cases, at the time of diagnosis and at 12 and 24 months during the follow-up period:

a) Health-related quality of life

b) Satisfaction with the quality of the hospital-based care

c) Functional status

#### Secondary objectives

To identify those factors affecting colorectal cancer patients’ quality of life, satisfaction with the hospital-based care, and their functional status. In particular:

a) To determine whether diagnosis delay, treatment delay and different follow-up strategies influence patients’ quality of life and satisfaction with the quality of the hospital-based care.

b) To determine if patients’ comorbidity and TNM stage at diagnosis are associated with quality of life and functional status in patients with colorectal cancer.

c) To determine the impact of colostomy on quality of life in patients with colorectal cancer.

## Methods/design

### Design

Single-centre prospective follow-up study of colorectal cancer patients diagnosed at the Complexo Hospitalario Universitario A Coruña (A Coruña, Spain) during the period 2011–2012. Incident cases with anatomopathological confirmation of colorectal cancer according to the International Classification of Diseases (ICD) 9th revision (codes 153 and 154) are included. Prevalent or recurrent cases are excluded, together with cases of multiple cancers, cases that are only dealt with in private hospitals, cases detected through colorectal cancer screening, and cases diagnosed in another hospital but which are referred for treatment to the hospital where the study is conducted.

### Data collection

Eligible patients are identified from the Department of Pathology within one month of diagnosis. At an initial interview with a trained nurse, patients are informed about the aims and characteristics of the study. After signing the Informed Consent Form*,* information is obtained from patients regarding their sociodemographic characteristics, family history of cancer, first symptoms attributable to cancer perceived by the patient, symptom perception and reaction to early symptoms. At the same time, patients are asked to complete the questionnaires concerning their health-related quality of life and satisfaction with hospital care. The patients’ functional impairment is also evaluated using Karnofsky Performance Status.

In the case of patients qualifying for curative surgical treatment, the initial interview is performed in the month following discharge after surgery. Patients subjected only to palliative procedures are interviewed within three months of the diagnosis. All of them will be followed-up for a maximum of 2 years, until December 2013. For survivors, health-related quality of life and functional status assessments will be re-evaluated by means of a personal interview with a trained nurse at one and two years after the diagnosis during the study period.

Clinical records are also reviewed to collect information about comorbidity at diagnosis (Charlson comorbidity index), tumour characteristics at diagnosis (site according to ICD-9th, tumour size, histological grade, TNM stage, location of metastases and infiltration of adjacent organs), treatment received (surgery, chemotherapy and/or radiation therapy), hospital consultations related to the colorectal cancer and exploratory procedures in the follow-up. Occurrence of the following incidents in the follow-up is also registered: local recurrence, development of metastases, appearance of a new tumour, and mortality.

Symptoms-to-diagnosis interval is defined as the time elapsed from the date the patient perceived the first symptoms until the cytohistological confirmation of the diagnosis of cancer (date of biopsy or direct surgery). Treatment delay is defined as the interval from date of diagnosis to date of treatment (i.e., date of surgery, date of preoperative or postoperative radiotherapy or chemotherapy or date of palliative treatment). For those patients who do not receive any treatment, the date of the visit closest to the decision not to treat the patient will be recorded.

Surveillance strategies will be classified based on the frequency of follow-up and the investigations performed, taking into account: a) history and physical examinations, b) serum carcinoembryonic antigen (CEA) determinations, c) imaging examinations (ecographies, positron emission tomographies, computed tomographies, magnetic resonances), and d) endoscopy explorations (colonoscopies or rectoscopies).

### Health-related quality of life

Health-related quality of life, the main outcome of this study, is assessed with the Quality of Life Questionnaire Core 30 (QLQ-C30) (version 3.0) [8] and the colorectal cancer-specific quality of life questionnaire module (QLQ*-*CR29) [35], developed by the European Organisation for Research and Treatment of Cancer (EORTC). These questionnaires have been translated and validated for use in Spain [36,37].

The EORTC QLQ-C30 is a generic cancer health-related quality of life questionnaire, which has to be supplemented by disease-specific modules for each type of cancer or tumour location*.* The EORTC QLQ-C30 consists of 30 items and includes a scale measuring the global health status/health-related quality of life, 5 functioning scales (physical, role, emotional, cognitive and social), 3 symptom scales (fatigue, nausea/vomiting and pain), and 6 single-item scales (dyspnea, insomnia, appetite loss, constipation, diarrhoea and financial impact).

The QLQ-CR29 is a colorectal cancer disease–specific module consisting of 29 items. These are grouped into 4 functioning scales (body image, anxiety, weight and sexual interest) and 18 symptom scales (urinary symptoms, stool characteristics, gastrointestinal symptoms, pain and other symptomatology) [9]. Items related to gastrointestinal symptoms are collected differently in stoma and non-stoma patients. Problems associated with sexual activity are evaluated differently for men and women, with a separate item for each gender.

The items on both questionnaires will be scaled and scored using the recommended EORTC procedures [38]. After estimating the average of the items that contribute to each scale, raw scores will be linearly transformed to a scale ranging from 0 to 100, with a higher score representing a higher (better) level of functioning or a higher (worse) level of symptoms. According to EORTC scoring guidelines, this procedure is only applicable to those patients with at least half of the items answered for each of the scales. In that case, any items with missing values will be ignored when making the calculations.

### Satisfaction with hospital-based care

Patient satisfaction with care is assessed by means of the validated Spanish version of the EORTC IN-PATSAT32 questionnaire [30,31]. This questionnaire was developed by the EORTC Quality of Life Group for assessing cancer patients’ perception of the quality of hospital-based care.

The EORTC IN-PATSAT32 is composed of 32 items organised into eleven multi-item scales and three single items assessing cancer patients’ perception of the quality of hospital doctors and nurses, as well as selected aspects of the care organisation and hospital environment. Each question is administered using a 5-category Likert response scale (“poor”, “fair”, “good”, “very good” or “excellent”).

The dimensions measured include doctors’ and nurses’ technical skills, interpersonal skills, information provision, and availability; satisfaction with other hospital staff interpersonal skills and information provision; exchange of information with the care team; waiting-time; hospital access; hospital comfort; and overall satisfaction with care. Following the standard scoring procedure for the EORTC IN-PATSAT32, all scores will be linearly transformed to a 0–100 scale, with higher scores reflecting a higher level of satisfaction.

### Functional status

Functional status of colorectal cancer patients is assessed by means of the Karnofsky Performance Status Scale (KPS) [39]. The KPS is an 11-point rating scale which ranges from normal functioning (100) to dead (0), used to assess patients’ physical functional level related to cancer and its treatment.

### Sample size

Sample size is limited by both the duration of the study and the number of incident colorectal cancer patients diagnosed per year. During the period 2011–2012 approximately n = 500 patients will be diagnosed of colorectal cancer. Assuming a non-response rate of 25%, a sample size of n = 375 patients is expected.

Based on their 0–100 scoring system, and using the range rule of thumb, a standard deviation of 25 points (one quarter of the range score) is assumed for the final punctuations of the EORTC QLQ-C30, QLQ-CR29 and IN-PATSAT32 questionnaires. Therefore, a sample size of n = 375 patients will allow us to estimate mean values with a precision of ±2.6 for a 95% confidence interval. Working with a power of 80% and an alpha of 0.05, score differences of 8 points between groups of patients will be detected as statistically significant, assuming an exposure of 50% to the variable of interest. This sample size will also allow detecting as statistically significant (p ≤ 0.05) correlation coefficients ≥0.15 among the studied variables and the questionnaires scores.

### Statistical analysis

Descriptive analyses will be performed for all variables. Continuous variables will be reported using means ± standard deviations (SD) or median (interquartile range). For dichotomous/categorical variables, absolute numbers and percentages will be computed, together with their 95% confidence intervals.

Health-related quality of life, satisfaction with care and functional status scores will be compared according to patients’ characteristics and disease variables. The comparison of means will be carried out using Student’s t test, Mann–Whitney test, analysis of variance or the Kruskall-Wallis test as appropriate. The association of qualitative variables will be carried out using Chi-square statistics. The correlation among quantitative variables will be assessed using Spearman’s Rho correlation coefficient, due to the expected non-normal distribution of the questionnaires scores and in order to detect nonlinear relatioships.

In the multivariate analysis, multiple linear and logistic regression models will be used to identify those variables independently associated with patients’ functional status after the diagnosis, quality of life and satisfaction with care scores. Separate regressions will be conducted for each of the three outcomes. Box-Cox normalizing transformations will be used when necessary to ensure the normality assumption in the linear regression model.

Finally, evolution of quality of life at one and two years from diagnosis will be analysed based on the change from baseline scores for each time point. Significance of the changes will be assessed using the Wilcoxon signed ranks test. Clinical relevance will be analyzed by means of the minimal important difference (MID), effect sized, and standard error of measurement (SEM). Information provided by the developers of the EORTC QLQ-C30 about what could be considered as a MID will be used. Therefore, a mean change score of 5 to 10 will be a “little” change, about 10 to 20 a “moderate” change, and more than 20 a large change. Cohen’s guidelines for interpreting small (effect size 0.2-0.49), moderate (effect size 0.5-0.79), and large (effect size ≥0.5) will also be considered. Finally, a value greater than 1 SEM will be considered as clinically significant.

Since measurements of QOL will be made repeatedly on the same patients, we will be in a repeated measures context. Longitudinal QOL will be analysed with a linear mixed-effects regression model. More specifically, the relationship of QOL with time will be determined using a random coefficients model, which generalizes linear regression techniques to allow for repeated observations. These models take into account the correlation within observations on the same subject and allow for inclusion of data on subjects who have only partial follow-up without imputing missing data.

Therefore, a linear random coefficients regression model will be performed, with QOL scores over time as the dependent variable. The relationship between QOL and time will be modeled by including a quantitative time effect (months since diagnosis) as a covariate in the model, fitting patient effect and patient*time interaction as random effects. In addition to the effect of time on the QOL outcome measures, we will incorporate other factors into the regression models, in order to adjust for those variables of interest and potential confounding factors such as: sociodemographic variables, diagnosis/treatment delay, follow-up strategies, comorbidy, TNM stage at diagnosis, or presence of colostomy.

Two-sided tests will be used, and p-values < 0.05 will be considered as statistically significant. Statistical analyses will be performed using SPSS for Windows (version 19.0, SPSS Inc., Chicago, Illinois) and R (version 2.12.2). Apart from performing an analysis of all of the patients, the patients with colon cancer and patients with rectal cancer will be analysed independently.

### Ethics

The study will be carried out according to the Good Clinical Practice guidelines of the Helsinki declaration. Informed consent is obtained from each patient to take part in the study and to review their clinical records. This project was approved by the corresponding ethics review board (Clinical Research Ethical Committee of Galicia, decision 2010/304).

## Discussion

Health-related quality of life, as well as other patient-related outcomes, is now considered an important endpoint in oncology studies [5]. Measurement of quality of life in colorectal cancer patients provides valuable information regarding the burden of the disease and side effects of cancer therapies. In addition, there is also evidence that a better QOL is associated with a prolonged survival of patients with cancer [7]. Therefore, improving the quality of life of these patients may lead to an improvement of their prognosis.

Understanding the characteristics associated to quality of life may help clinicians to identify patients at risk for poor quality of life, as well as to plan medical, psychological or social interventions to improve the patient’s well-being. Gender, age, income, education level and social network have been identified as general determinants of quality of life in colorectal cancer survivors. Other health-related factors, such as a higher body mass index or having more comorbidities also seems to be associated to poorer quality of life. Among cancer-related factors, no association was found with stage, while few studies have evaluated the relationship between different treatment regimens and quality of life [29].

Inconclusive results were observed concerning the effects of a permanent colostomy on quality of life [29,40]. In this sense, a Cochrane review comparing the quality of life in patients with or without permanent colostomy showed that it was not possible to draw conclusions whether the quality of life of stoma patients is poorer than that of non-stoma patients or not [40].

Information about patient satisfaction is also important in assessing the quality of health services. Studying the satisfaction of oncologic patients is especially important, because of the complexity of their treatments and the intensive monitoring they usually undergo. Until now, few studies have assessed the satisfaction of cancer patients by means of a validated instrument like the EORTC IN-PATSAT32 [30,36,41,42]. Published studies include oncologic patients with different locations, and we have not found any that only refer to colorectal cancer patients [30,36,41,42].

Although it is popularly assumed that delay has a significant and harmful impact on survival, recent reviews suggest that there is no association between diagnostic or therapeutic delays and prognosis in colorectal cancer patients [43,44]. Some studies even found counterintuitive results, showing that patients with short diagnostic intervals had higher mortality rates than patients with long diagnostic intervals [43]. This is the so-called “waiting time paradox,” and has also been reported for other types of cancer [45].

Quality of life and patient satisfaction present an alternative way for evaluating consequences of experiencing delay among these patients. Since a recent study has found a negative association between diagnostic delay and quality of life and patient satisfaction in gynaecologic cancer patients [34], we may hypothesize that long diagnosis delay could be an indicator of poorer quality of life and lower satisfaction levels in colorectal cancer patients.

This study will prospectively determine the quality of life of patients with colorectal cancer, and related variables. Its results will allow a deeper understanding of the impact of the disease from a more patient-centred approach. It will also allow us to recognize significant quality-of-life predictors, and as a result, to identify subgroups of patients with special needs. We will also determine the satisfaction of colorectal cancer patients with their hospital-based care, as well as potential predictors of their satisfaction level. By identifying background or organisational factors associated with patient satisfaction, it will be possible to determine groups of patients in need of additional attention, as well as areas for improvement. Special attention will be given to the relationship between diagnostic or therapeutic delay and patients’ quality of life and satisfaction levels.

The key strengths of this study include its longitudinal prospective design, the use of well-established instruments, and the large amount of information recorded from each patient. However, the results could be limited by the response rate and the proportion of participants who do not complete all of the assessments. The possibility of non-random dropout is also an important methodological issue. Evidently, the people who did not respond to the questionnaires may be different to those who did. Non-responders and those who die during follow-up could have lower quality of life than responders; therefore the reported QOL could overestimate the QOL of patients with colorectal cancer. The opposite could also occur, if patients with a good quality of life decide not to respond. Moreover, non-response may also affect the identification of predictors of QOL and satisfaction levels. We will try to maximize the response rate by performing a personal interview with each patient, in which QOL and satisfaction with care questionnaires will be completed.

Other limitations could be the single-centre nature and short study duration. Although single-centre studies simplify data collection and typically deal with a less heterogeneous population (therefore diminishing confounding), they have potentially limited external validity. A multicentre study could be more efficient and would have the advantage of increased generalizability of the results. On the other hand, this study will only analyse the early impact of colorectal cancer on the patients’ quality of life, in the first and second year after diagnosis. Additional follow-up will be required to investigate long-term QOL and related variables.

## Abbreviations

EORTC: European organisation for research and treatment of cancer; FACT-C: Functional assessment of cancer therapy-colorectal; ICD: International classification of diseases; KPS: Karnofsky performance status scale; QOL: Quality of life; SD: Standard deviation.

## Competing interests

The authors declare that they have no competing interests.

## Authors’ contributions

SPF, SPD, BLC, TSP and EGG participated in the design and coordination of the study. SPD, TSP and BLC are the biostatisticians of the study. SPF, SPD, BLC, TSP, EGG, RSB, PGS and APS reviewed the study protocol and made suggestions that improved the design. All of these individuals are involved in the management of the study. SPF and SPD drafted the manuscript. All of the authors read, revised and approved the final manuscript.

## References

[B1] Jemal A Bray F Center MM Ferlay J Ward E Forman D Global cancer statistics CA Cancer J Clin 2011 61 2 69 90 21296855 10.3322/caac.20107

[B2] Center MM Jemal A Smith RA Ward E Worldwide variations in colorectal cancer CA Cancer J Clin 2009 59 6 366 378 19897840 10.3322/caac.20038

[B3] Edwards BK Ward E Kohler BA Eheman C Zauber AG Anderson RN Jemal A Schymura MJ Lansdorp-Vogelaar I Seeff LC Annual report to the nation on the status of cancer, 1975–2006, featuring colorectal cancer trends and impact of interventions (risk factors, screening, and treatment) to reduce future rates Cancer 2010 116 3 544 573 19998273 10.1002/cncr.24760 PMC3619726

[B4] Brenner H Francisci S de Angelis R Marcos-Gragera R Verdecchia A Gatta G Allemani C Ciccolallo L Coleman M Sant M Long-term survival expectations of cancer patients in Europe in 2000–2002 Eur J Cancer 2009 45 6 1028 1041 19091549 10.1016/j.ejca.2008.11.005

[B5] Outcomes of cancer treatment for technology assessment and cancer treatment guidelines American society of clinical oncology J Clin Oncol 1996 14 2 671 679 8636786 10.1200/JCO.1996.14.2.671

[B6] Malhotra V Basow DS Functional problems in the patient with cancer UpToDate 2012 Waltham, MA: UpToDate

[B7] Montazeri A Quality of life data as prognostic indicators of survival in cancer patients: an overview of the literature from 1982 to 2008 Health Qual Life Outcomes 2009 7 102 20030832 10.1186/1477-7525-7-102 PMC2805623

[B8] Aaronson NK Ahmedzai S Bergman B Bullinger M Cull A Duez NJ Filiberti A Flechtner H Fleishman SB de Haes JC The European organization for research and treatment of cancer QLQ-C30: a quality-of-life instrument for use in international clinical trials in oncology J Natl Cancer Inst 1993 85 5 365 376 8433390 10.1093/jnci/85.5.365

[B9] Whistance RN Conroy T Chie W Costantini A Sezer O Koller M Johnson CD Pilkington SA Arraras J Ben-Josef E Clinical and psychometric validation of the EORTC QLQ-CR29 questionnaire module to assess health-related quality of life in patients with colorectal cancer Eur J Cancer 2009 45 17 3017 3026 19765978 10.1016/j.ejca.2009.08.014

[B10] Ward WL Hahn EA Mo F Hernandez L Tulsky DS Cella D Reliability and validity of the functional assessment of cancer therapy-colorectal (FACT-C) quality of life instrument Qual Life Res 1999 8 3 181 195 10472150 10.1023/a:1008821826499

[B11] Pucciarelli S Del Bianco P Efficace F Serpentini S Capirci C De Paoli A Amato A Cuicchi D Nitti D Patient-reported outcomes after neoadjuvant chemoradiotherapy for rectal cancer: a multicenter prospective observational study Ann Surg 2011 253 1 71 77 21135694 10.1097/SLA.0b013e3181fcb856

[B12] Wilson TR Alexander DJ Kind P Measurement of health-related quality of life in the early follow-up of colon and rectal cancer Dis Colon Rectum 2006 49 11 1692 1702 17041750 10.1007/s10350-006-0709-9

[B13] Peng J Shi D Goodman KA Goldstein D Xiao C Guan Z Cai S Early results of quality of life for curatively treated rectal cancers in Chinese patients with EORTC QLQ-CR29 Radiat Oncol 2011 6 93 21835046 10.1186/1748-717X-6-93 PMC3177872

[B14] Yost KJ Hahn EA Zaslavsky AM Ayanian JZ West DW Predictors of health-related quality of life in patients with colorectal cancer Health Qual Life Outcomes 2008 6 66 18724874 10.1186/1477-7525-6-66 PMC2538505

[B15] Smith-Gagen J Cress RD Drake CM Romano PS Yost KJ Ayanian JZ Quality-of-life and surgical treatments for rectal cancer–a longitudinal analysis using the California cancer registry Psychooncology 2010 19 8 870 878 19862692 10.1002/pon.1643 PMC2911491

[B16] Gall CA Weller D Esterman A Pilotto L McGorm K Hammett Z Wattchow D Patient satisfaction and health-related quality of life after treatment for colon cancer Dis Colon Rectum 2007 50 6 801 809 17285234 10.1007/s10350-006-0815-8

[B17] Ramsey SD Andersen MR Etzioni R Moinpour C Peacock S Potosky A Urban N Quality of life in survivors of colorectal carcinoma Cancer 2000 88 6 1294 1303 10717609

[B18] Arndt V Merx H Stegmaier C Ziegler H Brenner H Restrictions in quality of life in colorectal cancer patients over three years after diagnosis: a population based study Eur J Cancer 2006 42 12 1848 1857 16829069 10.1016/j.ejca.2006.01.059

[B19] Domati F Rossi G Benatti P Roncucci L Cirilli C Ponz de Leon M Long-term survey of patients with curable colorectal cancer with specific reference to the quality of life Intern Emerg Med 2011 6 6 529 535 21512795 10.1007/s11739-011-0590-y

[B20] Jansen L Herrmann A Stegmaier C Singer S Brenner H Arndt V Health-related quality of life during the 10 years after diagnosis of colorectal cancer: a population-based study J Clin Oncol 2011 29 24 3263 3269 21768465 10.1200/JCO.2010.31.4013

[B21] Chambers SK Meng X Youl P Aitken J Dunn J Baade P A five-year prospective study of quality of life after colorectal cancer Qual Life Res 2012 21 9 1551 1564 22200938 10.1007/s11136-011-0067-5

[B22] Ramsey SD Berry K Moinpour C Giedzinska A Andersen MR Quality of life in long term survivors of colorectal cancer Am J Gastroenterol 2002 97 5 1228 1234 12017152 10.1111/j.1572-0241.2002.05694.x

[B23] Trentham-Dietz A Remington PL Moinpour CM Hampton JM Sapp AL Newcomb PA Health-related quality of life in female long-term colorectal cancer survivors Oncologist 2003 8 4 342 349 12897331 10.1634/theoncologist.8-4-342

[B24] Pollack J Holm T Cedermark B Altman D Holmström B Glimelius B Mellgren A Late adverse effects of short-course preoperative radiotherapy in rectal cancer Br J Surg 2006 93 12 1519 1525 17054311 10.1002/bjs.5525

[B25] Phipps E Braitman LE Stites S Leighton JC Quality of life and symptom attribution in long-term colon cancer survivors J Eval Clin Pract 2008 14 2 254 258 18284521 10.1111/j.1365-2753.2007.00842.x

[B26] Krouse RS Herrinton LJ Grant M Wendel CS Green SB Mohler MJ Baldwin CM McMullen CK Rawl SM Matayoshi E Health-related quality of life among long-term rectal cancer survivors with an ostomy: manifestations by sex J Clin Oncol 2009 27 28 4664 4670 19720920 10.1200/JCO.2008.20.9502 PMC2754912

[B27] Hamashima C Long-term quality of life of postoperative rectal cancer patients J Gastroenterol Hepatol 2002 17 5 571 576 12084031 10.1046/j.1440-1746.2002.02712.x

[B28] Fucini C Gattai R Urena C Bandettini L Elbetti C Quality of life among five-year survivors after treatment for very low rectal cancer with or without a permanent abdominal stoma Ann Surg Oncol 2008 15 4 1099 1106 18181002 10.1245/s10434-007-9748-2

[B29] Jansen L Koch L Brenner H Arndt V Quality of life among long-term (≥5 years) colorectal cancer survivors–systematic review Eur J Cancer 2010 46 16 2879 2888 20605090 10.1016/j.ejca.2010.06.010

[B30] Brédart A Bottomley A Blazeby JM Conroy T Coens C D’Haese S Chie WC Hammerlid E Arraras JI Efficace F An international prospective study of the EORTC cancer in-patient satisfaction with care measure (EORTC IN-PATSAT32) Eur J Cancer 2005 41 14 2120 2131 16182120 10.1016/j.ejca.2005.04.041

[B31] Arraras JI Vera R Martínez M Hernández B Laínez N Rico M Vila M Chicata V Asín G The EORTC cancer in-patient satisfaction with care questionnaire: EORTC IN-PATSAT32 Validation study for Spanish patients Clin Transl Oncol 2009 11 4 237 242 19380301 10.1007/s12094-009-0346-6

[B32] Esteva M Ramos M Cabeza E Llobera J Ruiz A Pita S Segura J Cortes J Gonzalez-Lujan L group Dr Factors influencing delay in the diagnosis of colorectal cancer: a study protocol BMC Cancer 2007 7 86 17697332 10.1186/1471-2407-7-86 PMC1894641

[B33] Fernandez SP Diaz SP Calvino BL Santamaria PG Pillado TS Monreal FA Macia F Calavera MAS Macias AE Ayerbes MV Diagnosis delay and follow-up strategies in colorectal cancer. Prognosis implications: a study protocol BMC Cancer 2010 10 6 20920369 10.1186/1471-2407-10-528 PMC2958943

[B34] Robinson KM Christensen KB Ottesen B Krasnik A Diagnostic delay, quality of life and patient satisfaction among women diagnosed with endometrial or ovarian cancer: a nationwide Danish study Qual Life Res 2012 21 9 1519 1525 22138966 10.1007/s11136-011-0077-3

[B35] Gujral S Conroy T Fleissner C Sezer O King PM Avery KN Sylvester P Koller M Sprangers MA Blazeby JM Assessing quality of life in patients with colorectal cancer: an update of the EORTC quality of life questionnaire Eur J Cancer 2007 43 10 1564 1573 17521904 10.1016/j.ejca.2007.04.005

[B36] Arraras JI Arias F Tejedor M Pruja E Marcos M Martínez E Valerdi J The EORTC QLQ-C30 (version 3.0) Quality of Life questionnaire: validation study for Spain with head and neck cancer patients Psychooncology 2002 11 3 249 256 12112486 10.1002/pon.555

[B37] Arraras J Villafranca E Arias F Dominguez M Lainez N Manterola A Martinez E Romero P Martinez M The EORTC Quality of Life Questionnaire QLQ-C30 (version 3.0). Validation study for Spanish prostate cancer patients Arch Esp Urol 2008 61 8 949 954 19040169 10.4321/s0004-06142008000800017

[B38] Aaronson NK Fayers PM Bjordal K Groenvold M Curran D Bottomley A The EORTC QLQC30 Scoring Manual 2001 3 Brussels: European Organisation for Research and Treatment of Cancer

[B39] Karnofsky D Burchenal J The clinical evaluation of chemotherapeutic agents in cancer Evaluation of Chemotherapeutic Agents 1949 New York: Columbia University Press

[B40] Pachler J Wille-Jørgensen P Quality of life after rectal resection for cancer, with or without permanent colostomy Cochrane Database Syst Rev 2005 2 CD004323 10.1002/14651858.CD004323.pub3 15846707

[B41] Balderas-Peña LM Sat-Muñoz D Contreras-Hernández I Solano-Murillo P Hernández-Chávez GA Mariscal-Ramírez I Lomelí-García M Díaz-Cortés MA Mould-Quevedo JF Castro-Cervantes JM Evaluation of patient satisfaction with the quality of health care received within the EORTC IN-PATSAT32 trial by patients with breast and colorectal cancer, and non-Hodgkin lymphoma at different stages. Correlation with sociodemographic characteristics, comorbidities and other procedural variables at the Mexican Institute of Social Security Value Health 2011 14 5 Suppl 1 S96 S99 21839910 10.1016/j.jval.2011.05.026

[B42] Brédart A Coens C Aaronson N Chie WC Efficace F Conroy T Blazeby JM Hammerlid E Costantini M Joly F Determinants of patient satisfaction in oncology settings from European and Asian countries: preliminary results based on the EORTC IN-PATSAT32 questionnaire Eur J Cancer 2007 43 2 323 330 17156997 10.1016/j.ejca.2006.10.016

[B43] Ramos M Esteva M Cabeza E Campillo C Llobera J Aguiló A Relationship of diagnostic and therapeutic delay with survival in colorectal cancer: a review Eur J Cancer 2007 43 17 2467 2478 17931854 10.1016/j.ejca.2007.08.023

[B44] Ramos M Esteva M Cabeza E Llobera J Ruiz A Lack of association between diagnostic and therapeutic delay and stage of colorectal cancer Eur J Cancer 2008 44 4 510 521 18272362 10.1016/j.ejca.2008.01.011

[B45] Neal RD Do diagnostic delays in cancer matter? Br J Cancer 2009 101 Suppl 2 S9 S12 19956171 10.1038/sj.bjc.6605384 PMC2790696

